# Prevalence of Hepatitis C infection in Qeshm Island in 2013-2014, Iran

**Published:** 2015

**Authors:** I Ghasemzadeh, A Alavi-Nasr, M Khademi, A Kargar Kheirabad, H Gouklani

**Affiliations:** * Infectious and Tropical Diseases Research Center, Hormozgan University of Medical Sciences, Bandar Abbas, Iran,; ** Clinical Research Development Center, Shahid Mohammadi Hospital, Hormozgan University of Medical Sciences, Bandar Abbas, Iran,; *** Department of Virology, School of Public Health, Tehran University of Medical Sciences, Tehran, Iran,; **** Molecular Medicine Research Center, Hormozgan University of Medical Sciences, Bandar Abbas, Iran

**Keywords:** epidemiology, Hepatitis C, Qeshm

## Abstract

**Introduction:** Hepatitis has involved many individuals and has left many complications. Hepatitis C is a type of hepatitis connected with several dilemmas.

The purpose of the research is to study the Hepatitis epidemiology C into the Island of Qeshm in 2014.

**Method:** this was an interventional study conducted on 1500 inhabitants of Qeshm Island. Participants were selected by using cluster sampling. Five cc of blood was drawn from each participant in order to test for HCV-Ab with ELIZA technique. Positive samples were referred for PCR to investigate the presence of anti Hepatitis C anti body. Data were entered in SPSS v.16 after sample collection and are examined utilizing detailed census (prevalence, mean, percent and standard deviation) and chi-square.

**Results:** out of 1500 participants, 986 (65.7%) are women and 514 (34.3 %) are men. HCV anti body was seen in four patients (0.3 percent). The outcomes of the research explained that not of the studied factors (age, gender, marital status, place of residence, educational level, history of IV drug abuse, being in jail, quitting addiction, risky sexual behavior, etc.) is related to antibody pervasiveness.

**Conclusion:** The disease pervasiveness was 0.3 percent in Qeshm Island, that is compatible with the another research outcomes. Also, factors investigated for HCV were not recognized as HCV risk factors.

## Introduction

Viruses are present everywhere and have important roles in health and evolution. Many biologists recognize them as creatures with specific and various living areas that can swap genes between different species [**1**].

Hepatitis is a transmissible illness which causes inflammation in the liver [**2**]. This disease is classified to Hepatitis B, A, E and C [**3**-**6**].

Hepatitis C is an inflammatory and liver necrosis disease, which develops as acute or chronic and belongs to the flaviviridae family. It tends to live inside liver to proliferate among its tissue. This virus include single stranded positive RNA with 9500 nucleotides [**7**]. Genotype one Hepatitis C virus consists of 60 to 75 percent of positive HCV cases in the USA. The treatment of that sort is the very hard therapy [**8**].

Hepatitis C has different complications including neurological complications. That can too cause hemorrhagic or ischemic CVA. Thus, HCV must be recognized via a differential diagnosis for neurological disturbances [**9**]. However, that very significant complications was associated to the liver [**10**]. Chronic hepatitis is one of the other complications of this disease [**11**]. Some of those cases will be led to liver cirrhosis [**12**]. Another dangerous complication of Hepatitis C is forming hepatocellular carcinoma which can evolve two to four decades after the hepatitis infection [**13**].

The different prevalence of Hepatitis C was reported. The Scandinavian countries and England have the lowest prevalence [**14**]. Other researches have explained that this pervasiveness of Hepatitis C has increased from 2.5 percent in 1998 to 2.8 percent in 2005 and the number of those cases has changed from 122 million to 185 million, worldwide [**14**]. 

Since not past researches were managed in this area, this research is carried out to ascertain the pervasiveness of Hepatitis C among the common people of Qeshm Island is 2014.

## Method

The definitive research is directed in 2014 in Qeshm Island of Hormozgan province. The research society include 130.000 inhabitants. The sample size is determined to be 1500, by using the following formula:

$$n=\frac{z_{1-\frac{\alpha }{2}}^{2}p(1-p)}{d^{2}}$$

Before the research, the Ethics Committee of Hormozgan Medical Sciences University accepted this research. Stratified sampling is utilized for choose the participants. Qeshm was divided into several geographic regions and individuals who referred to health centers or hospitals were asked to fill in the checklist and be evaluated for HCV. Data were collected by utilizing a checklist that is created based on similar studies and the experts’ opinion. Data that was collected included name, surname, age, gender, occupation, marital status, residence status, educational level, ethnicity, religion, history of blood donation, knowledge regarding virus of Hepatiti C, how they acquired knowledge (friends and family, television, radio and television, newspapers, hospitals and medical centers), blood type and previous history diseases. Also, participants are ensured that each data will remain confidential. All participants provided an educated, signed permission. 

If a person providing a consent and live in Qeshm included to study, and person with cardiovascular disease, RF, Infections, recent measles infection, mumps, Infectious mononucleosis, past medical history of malaria, brucellosis, tuberculosis, other viral hepatitis, HIV/AIDS, toxoplama, GI problem, pregnancy, recent trauma, hematogenous or sexual transmitted disease, blood transfusion during one year ago, lactation, vaccination, previous immunoglobulin injection, psyachtric disease, diabetes mellitus also thyroid disorders excluded from study 

In the research, five cc of blood was drained from any contributor for HCV-Ab evaluation using a third generation anti-HCV kit (Biometrix). Positive samples (according to ELIZA method) were referred for PCR (third generation, made in France) to confirm the anti-HCV immunoglobulin’s presence. Patients with positive HCV RIBA were considered infected with Hepatitis C or having a HCV history. Another five cc of venous blood was drained from the brachial vein and the serum was isolated and evaluated to HCV according to ELIZA technique. Positive cases are defined and referred for genome extraction with RT- PCR utilizing special introductions for HCV infection. Data was entered in SPSS v.16 software and analyzed by expressive statistics (, frequencies, average, percent, and scale variation) and chi-square.

## Results

Among 1500 participants, 986 (65.7 percent) were females and 514 (34.3 percent) were males. Only four patients (0.3 percent) are established for HCV antibodies. 

The outcomes of the research explained that nothing of the examined factors was associated with the pervasiveness of the immunoglobulin. Two women (0.20 %) and two men (0.4 percent) had positive HCV antibodies and the difference of prevalence of HCV antibody was not important between kinds (p = 0.610).

Among all participants, 87.7 percent had an educational level of high-school diploma or below. All HCV positive patients were among them. But, no important relation was detected among HCV and the educational level (p=0.976).

In this study, 88 percent (1320 participants) were married and 12 percent (180 participants) were male. All HCV patients (four individuals) were married. There was not important relationship among the antibody prevalence and the conjugal state (p > 0.005).

In addition, 372 participants (24.8 percent) lived inside the urban area while 1128 (75.2 percent) lived in rural areas. All four patients are the village region. However, no significant association was seen between HCV antibodies and place of residence (p=0.578).

The mean age of HCV positive patients and the healthy group was 36.75 ± 16.78 years and 32.58 ± 13.16. This variation is not important (p=0.528).

No participant from the research had a records of IV drug abuse, being imprisoned, quitting addiction, or risky sexual behaviors. Where is no important relationship among those factors and the antibody pervasiveness (p>0.005). Also, all four patients had a history of dentist visits, however, this was not significant with the prevalence of antibody (p>0.005). Among all the participants of the study, one (0.07 percent) used opium and two (0.13 percent) used heroin. Neither the HCV positive patients was drug abusers. Where is no important relationship among drug abuse and HCV antibodies (p>0.005).

Also, no participant had a history of hemophilia, dialysis or organ transplantation.

## Conclusion

Hepatitis C infection is an important problem worldwide that affects almost 200 million people around the world [**15**]. In some countries, it is an essential cause of chronic liver disease and the very general cause of HCC [**16**,**17**]. The purpose of this research was to ascertain the pervasiveness of Hepatitis C between the common communities of Qeshm in 2014.

This was the first research directed in Qeshm Island that investigated the Hepatitis C pervasiveness. The outcomes explained that 0.3 percent of the members must HCV and the PCR results of four patients were positive. However, many types of research have been directed in Iran in this regard. 

Our result is compatible with the HCV pervasiveness among the common populace of Iran being below one percent in a research with Alavian et al. [**18**]. Alavian et al. [**19**] conducted another study and reported a prevalence of 0.16 percent and Merat [**20**] conducted another study which reported a prevalence of 0.5 percent in Iran. Other reports in Iran included: a pervasiveness of 0.83 percent reported by Sayad et al., 0.2 percent reported by Taghi Shakeri [**17**] and 0.05 percent reported by Zamani et al. in Amol [**21**]. The outcomes of our research are alike to the findings of other studies in Iran. The few differences can be related to several parts such as lifestyle, population density and education about Hepatitis C [**21**]. 

According to other studies, the HCV pervasiveness is larger in other countries; 5.9 percent in Hawaii [**22**], 2.7 percent among New York population aged above 20 [**23**], 1.57 percent in Pakistan [**24**], 1.71 percent in a state of Nigeria [**25**], 0.5 percent in Serbia and Tajikistan, 13 percent in Uzbekistan among the general population of WHO regions [**26**], 1.5 percent among Hispanic/ Latinos of America [**27**], two percent in Rhode Island of the US [**28**], 1.2 percent among the general population of Libya [**29**] and 4.62 percent in India [**30**].

Our study showed that half of the patients were males and half were females. Also, no association was detected among Hepatitis C and gender. Our outcome is compatible by Shakeri et al. [**17**] and Alavian et al. [**31**]. However, Sharifi et al. [**32**] and Veemehren et al. [**33**] and Thakral et al. reported inconsistent results and explained a larger Hepatitis C pervasiveness among the male population.

Although where was not important relationship seen between age and Hepatiti of C prevalence in our study, there were three cases in the 25-35 years age group. Shakeri et al. [**17**], Alavian et al. [**34**], Vermehre et al. [**33**] and Sharifi et al. [**32**] reported that older individuals are further inclined to this disease.

All four patients lived in rural regions of Qeshm. But, there was not important relationship among the position of hepatitis and residency. Fattahi et al. [**35**] reported a 0.24 percent prevalence in rural areas of Fars prevalence. Other researches have explained that the place of residence is no similar to Hepatitis C infection [**36**].

No participant of ours research had a previous history of hemophilia, using a shared needle, dialysis, or organ donation. Thus, we cannot discuss their effect on Hepatitis C. Other researches should describe them as Hepatitis C risk factors [**37**-**39**].

The prevalence of this disease was 0.3 percent in Qeshm Island, which is compatible with the outcomes of other studies, but lower than other world regions. It also proved a lower prevalence of Hepatitis C in Iran compared with another countries. 

**Limitations**

Some inhabitants of the region did not agree with the involvement in this study.

**Suggestions**

Other research should be directed to ascertain Hepatitis C genotype in Qeshm Island and investigate their complications.

**Acknowledgements**

The research is the result of a general physician thesis. We would relish to gratitude all professors, Qeshm inhabitants, health system authorities, and Hormozgan Medical Sciences University who helped us during this study.
